# The Metabolism of a Novel Cytochrome P450 (*CYP77B34*) in Tribenuron-Methyl-Resistant *Descurainia sophia* L. to Herbicides with Different Mode of Actions

**DOI:** 10.3390/ijms23105812

**Published:** 2022-05-22

**Authors:** Jing Shen, Qian Yang, Lubo Hao, Lingling Zhang, Xuefeng Li, Mingqi Zheng

**Affiliations:** Department of Applied Chemistry, College of Science, China Agricultural University, Beijing 100193, China; shenjingtust@cau.edu.cn (J.S.); yangq1991@163.com (Q.Y.); 13156971873@163.com (L.H.); zhangll@cau.edu.cn (L.Z.); lixuefeng2007@163.com (X.L.)

**Keywords:** flixweed, cytochrome P450 monooxygenases, metabolic resistance, resistance patterns

## Abstract

*Descurainia sophia* L. (flixweeds) is a noxious broad-leaf weed infesting winter wheat fields in China that has evolved high resistance to tribenuron-methyl. In this work, a brand new gene *CYP77B34* was cloned from tribenuron-methyl-resistant (TR) *D. sophia* and transferred into *Arabidopsis thaliana*, and the sensitivities of *Arabidopsis* with or without the *CYP77B34* transgene to herbicides with a different mode of actions (MoAs) were tested. Compared to *Arabidopsis* expressing pCAMBIA1302-GFP (empty plasmid), *Arabidopsis* transferring pCAMBIA1302-CYP77B34 (recombinant plasmid) became resistant to acetolactate synthase (ALS)-inhibiting herbicide tribenuron-methyl, protoporphyrinogen oxidase (PPO)-inhibiting herbicides carfentrazone-ethyl and oxyfluorfen. Cytochrome P450 inhibitor malathion could reverse the resistance to tribenuron-methyl, carfentrazone-ethyl and oxyfluorfen in transgenic *Arabidopsis* plants. In addition, the metabolic rates of tribenuron-methyl in *Arabidopsis* expressing *CYP77B34* were significantly higher than those in *Arabidopsis* expressing pCAMBIA1302-GFP. Other than that, the transgenic plants showed some tolerance to very-long-chain fatty acid synthesis (VLCFAs)-inhibiting herbicide pretilachlor and photosystem (PS) II-inhibiting herbicide bromoxynil. Subcellular localization revealed that the CYP77B34 protein was located in the endoplasmic reticulum (ER). These results clearly indicated that *CYP77B34* mediated *D. sophia* resistance to tribenuron-methyl and may have been involved in *D. sophia* cross-resistance to carfentrazone-ethyl, oxyfluorfen, pretilachlor and bromoxynil.

## 1. Introduction

Herbicides have been the most effective way for controlling weeds for several decades. Weeds have evolved serious resistance to a large number of herbicides due to extensive and intensive use of herbicides, which leads to serious loss of crop yields [1]. The mechanisms of weed resistance are typically classified into two broad categories. Target-site-based resistance (TSR) is endowed by gene mutation or deletion, or (and) gene overexpression or amplification of the herbicide target protein. Meanwhile, non-target-site-based resistance (NTSR) is achieved by enhanced herbicide metabolism (or) and sequestration, reduced penetration and (or) translocation [2,3]. In comparison with the well-known TSR, NTSR is focused on rarely and often covered by TSR or purposely ignored by researchers due to its complexities and difficulties. Metabolic resistance, one of the most common and important NTSRs, is caused by enhanced herbicide metabolism in weeds. Herbicide metabolism in plants usually experiences phases of conversion, conjugation and compartmentation, which are mediated by different metabolic enzymes. These metabolic enzymes include cytochrome P450 monooxygenase (P450s), glucosyltransferases (GTs), glutathione S-transferases (GSTs), aldo-keto reductase, ATP binding cassette transporters (ABC transporters) and esterases. Hence, enhanced metabolism of herbicides is one of the most common and important NTSR mechanisms [4,5,6,7].

Cytochrome P450 is one of the largest families in plant metabolism and represents around 1% of plant protein-coding genes. The P450s are regarded as the nature’s most versatile biological catalysts and catalyze numerous chemical reactions with countless substrates. These reactions refer to the typical mono-oxygenation process and atypical reactions of dimerization, isomerization, dehydration, etc. [8]. The P450s play crucial roles in the synthesis of various endogenous substances with important physiological functions in plants, such as hormones of brassinosteroid (BR), abscisic acid (ABA), gibberellin (GA) [9,10,11], signaling molecules of jasmonic acid (JA) and salicylic acid (SA) [12], plant defense molecules (suberin, lignin, flavonoids, phytoalexins) [13,14,15,16] and structural components (phenylpropanoids, fatty acids, sterols) [17,18]. In addition, P450s can metabolize all kinds of exogenous compounds including polychlorinated biphenyls (PCB), environmental chemicals, drugs and pesticides [8,19,20]. For this reason, P450s not only play important roles in plant growth, development and adaptation, but also are an excellent window to understanding plant evolution. Weed resistance is a good example of plant adaptation to herbicide selection pressures. Therefore, studies on the function of specific P450s are helpful not only to revealing the mechanisms of metabolic resistance in weeds, but also to understanding the evolutionary process of P450s.

*Descurainiasophia* L. (flixweeds) is a notorious broad-leaf weed infesting winter wheat fields in China that has evolved resistance to ALS-inhibiting herbicide tribenuron-methyl. Our previous work had confirmed that amino acid substitutions at positions Pro197 (substituted by Leu, Ser, Thr or Tyr), Asp376 (Glu) or Trp574 (Leu) in the ALS enzyme are the TSR mechanisms mainly responsible for *D. sophia* resistance to tribenuron-methyl [21,22,23]. The results of a P450 inhibitor and liquid chromatography−mass spectrometry (LC-MS) analysis confirmed that one or more P450 isoenzymes mediated *D. sophia* resistance to tribenuron-methyl [24]. Moreover, the results of the RNA-seq experiment indicated that metabolic enzyme families of GSTs, GTs and the ABC transporter may be involved in *D. sophia* resistance to tribenuron-methyl [25]. Based on the results of the above-mentioned research, five novel P450 genes with full-length sequences were identified and cloned from tribenuron-methyl-resistant (TR) *D. sophia*, which were named *CYP77B34* (NCBI accession number MF152623), *CYP81F5* (MF152624), *CYP709B8* (MF152625), *CYP96A146* (MF152630) and *CYP96A147* (MF152626) by the P450 nomenclature committee [24,25]. Although indirect evidence suggested that *CYP96A146* and *CYP96A147* may mediate *D. sophia* resistance to tribenuron-methyl, there is no direct evidence on these P450 being involved in *D. sophia* resistance to tribenuron-methyl [24]. In this work, the TR *D. sophia CYP77B34* was transferred into *Arabidopsis thaliana*, and the sensitivity of *A. thaliana* transformed with or without *CYP77B34* to herbicides with different MoAs was tested. Synergism of the P450 inhibitor malathion and metabolism analysis of tribenuron-methyl by LC-MS were used to further validate the roles of *CYP77B34* in herbicide resistance. Subcellular localization of CYP77B34 in *Nicotiana benthamiana* was observed. In addition, the expression levels of *CYP77B34* in *D. sophia* were determined before and after tribenuron-methyl treatment.

## 2. Results

### 2.1. Cloning and Characterization of the CYP77B34 Gene

The *CYP77B34* gene with full-length sequence was successfully cloned from TR *D. sophia* (N11) plants (Accession number MF152623) and contained a 1530 bp open reading frame (ORF) encoding a 510 amino acid protein. The deduced molecular weight of CYP77B34 was 57.8 kDa, and the theoretical pI was 8.58. The amino acid sequence of CYP77B34 had typical P450 conserved domains, such as the most diagnostic signature motif (F-x-x-G-x-R-x-C-x-G/A) (where x could represent any amino acid) for a P450 protein surrounding the heme cysteine ligand, less conserved sequences of Helix C (W-x-x-x-R), Helix I (A-G-x-D-T-S), Helix K (E-x-x-R) and meander (P-x-x-F-x-P-x-x-F). These typical structural domains indicated that *CYP77B34* was a member of P450 proteins (Figure S1). In addition, *CYP77B34* displayed high identity with *CYP77A3* in *Brassia napus* (73.95%), *CYP77B1* in *A. thaliana* (72.56%), *CYP77A3* in *Eutrema salsugineum* (77.67%) and *CYP77B1-1* in *Isatis tinctoria* (90.54%) (Figure S2).

The results of phylogenetic analysis indicated that *CYP77B34* showed specific orthologous relationships with other CYP77A and CYP77B subfamilies genes in different cruciferous plants, including *I. tinctoria*, *B. napus*, *Brassica rapa*, *Raphanus sativus*, *E. salsugineum*, *Camelina sativa*, *Capsella rubella*, *Arabidopsis lyrate* and *A. thaliana* (Figure S3).

### 2.2. Transcript Levels of the CYP77B34 Gene in D. sophia

**Figure 1:**
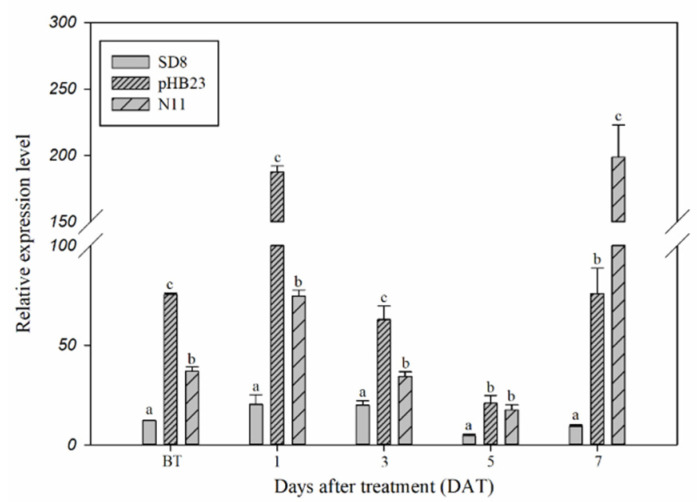
Expression levels of *CYP77B34* in TS (SD8) and TR (pHB23 and N11) *D. sophia* plants before (BT) or 1, 3, 5 and 7 days after treatment (DAT) with or without tribenuron-methyl. 18sRNA was the reference gene. All data are mean ± SE of three replicates. Columns with different letters indicate that the relative expression level had a significant difference at the same time point (using one-way analysis of variance, combined with Dunnett’s post-test comparison, *p* < 0.05).

Constitutive and inducible expression of the *CYP77B34* gene in *D. sophia* were determined in order to explore its roles in *D. sophia* resistance to tribenuron-methyl (Figure 1). The expression level of *CYP77B34* in pHB23 and N11 *D. sophia* populations was 6.21- and 2.16-fold higher, respectively, than that in tribenuron-methyl-susceptible (TS) *D. sophia* (SD8) before tribenuron-methyl treatment (BT). In addition, *CYP77B34* could be induced by tribenuron-methyl, and the expression level in TR *D. sophia* was always higher than that in TS *D. sophia* at 1, 3, 5 and 7 days after treatment (DAT). The expression levels increased about 9.00-fold in pHB23 at 1 DAT and 21.25-fold in N11 populations at 7 DAT comparing with that of TS *D. sophia*. Other than that, we also noted that the expression levels of *CYP77B34* in TS and TR *D. sophia* decreased at 3 and 5 DAT comparing with that at 1 DAT. This may be due to the enhanced concentration of tribenuron-methyl absorption by plants at 3 and 5 DAT, which inhibited the expression of *CYP77B34* TS and TR *D. sophia*. With the metabolism of tribenuron-methyl in two TR *D. sophia*, the inhibiting effect of tribenuron-methyl on *CYP77B34* expression disappeared and even exhibited an inducing effect on *CYP77B34* expression at 7 DAT.

### 2.3. Subcellular Localization of the CYP77B34 Protein

**Figure 2:**
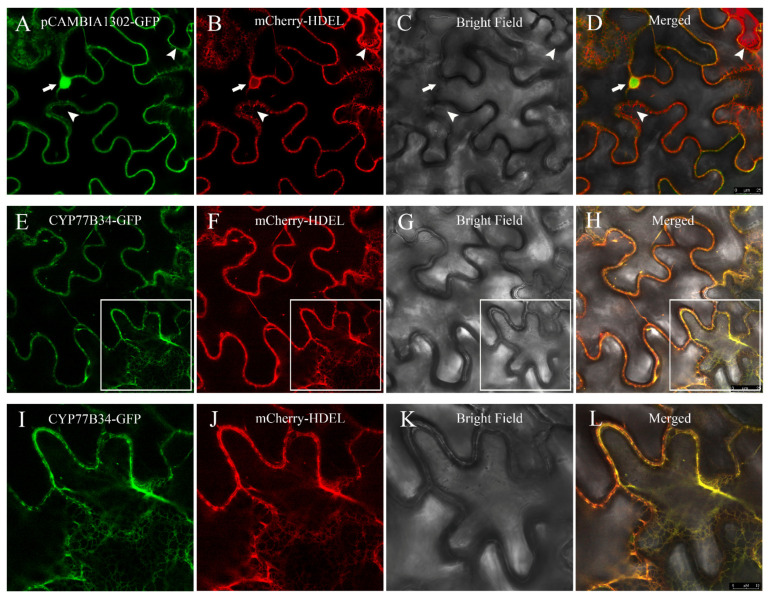
Subcellular localization of CYP77B34. In (**A**–**D**), pCAMBIA1302-GFP shown in green (**A**) co-expressed with the endoplasmic reticulum (ER) marker mCherry-HDEL shown in red; (**B**–**D**) are the bright-field and merged images, respectively. The arrows and arrowheads show the nucleus and the reticulate-structure ER. In (**E**–**H**), CYP77B34-GFP shown in green (**E**) co-expressed with mCherry-HDEL shown in red; (**F**–**H**) are the bright-field and merged images, respectively. Images (**I**–**L**) are the magnifications of the selected areas in (**E**–**H**). Scale bars: 25 μm in (**A**–**H**) and 10 μm in (**I**–**L**).

The results of transient expression indicated that the green fluorescence associated with the empty vector pCAMBIA1302-GFP distributed throughout the nucleus and cytoplasm (Figure 2A), and the red fluorescence emitted by the endoplasmic reticulum (ER) marker mCherry-HDEL was observed in the ER (Figure 2B). Merging of the green and red fluorescence was not unanimous, which suggested that the empty vector pCAMBIA1302-GFP was localized in the cytoplasm and the nucleus rather than in the ER (Figure 2D). When pCAMBIA1302-CYP77B34-GFP and mCherry-HDEL constructs were co-infiltrated in *N. benthamiana* leaves, the co-localization signals were observed in the ER network (Figure 2E–H). Pearson’s correlation coefficients between mCherry-HDEL and CYP77B34-GFP or mCherry-HDEL and pCAMBIA1302-GFP were 0.84 ± 0.021 and 0.27 ± 0.067, respectively (Figure S4). These results demonstrated that CYP77B34 was localized in the ER, which is similar to most P450s.

### 2.4. Herbicide Sensitivity of Transgenic Arabidopsis Expressing CYP77B34

In order to characterize the metabolic ability of *CYP77B34*, the herbicide sensitivities of transgenic *Arabidopsis* expressing CYP77B34-GFP or GFP were compared. Four individual transgenic lines were obtained with different transcript levels of *CYP77B34* (Figure S5). Obviously, the transgenic *Arabidopsis* carrying CYP77B34-GFP displayed different tolerance of herbicides with different MoAs. Compared with *Arabidopsis* expressing GFP, three lines of *Arabidopsis* carrying CYP77B34-GFP exhibited higher tolerance to tribenuron-methyl (ALS), carfentrazone-ethyl (PPO) and oxyfluorfen (PPO) (Table 1, Figures S6 and S7).

Among the three lines, the line of 77B34#7 displayed the highest tolerance to tribenuron-methyl, carfentrazone-ethyl and oxyfluorfen with RIs of 3.08, 1.82 and 2.13, respectively. In addition, *Arabidopsis* with CYP77B34-GFP had tolerance of herbicides of bromoxynil (PSII) and pretilachlor (ALCFAs) (Figure S7F,G). Most seedlings of *Arabidopsis* with GFP grown in 20 μM bromoxynil were etiolated, while there were few etiolated seedlings for *Arabidopsis* with CYP77B34-GFP (Figure S7F). The seedlings’ growth of *Arabidopsis* carrying GFP was completely inhibited by pretilachlor at the concentration of 80 μM, while that of the *Arabidopsis* carrying CYP77B34-GFP was much better (Figure S7G).

In addition, the sensitivities of *Arabidopsis* with or without the *CYP77B34* gene to other herbicides with different concentrations were tested in this work. These herbicides (MoAs) included imazamox and flumetsulam (ALS), napropamide (unknown MoA), glyphosate (enolpyruvyl shikimate phosphate synthase, EPSPS), glufosinate ammonium (glutamine synthetase, GS), diclofop-methyl (acetyl-coenzyme A carboxylase, ACCase), atrazine (PSII), propyzamide and pendimethalin (microtubule assembly), fluroxypyr (auxin mimics) and fomesafen (PPO). However, *Arabidopsis* with or without *CYP77B34* gene showed no obvious differences to the herbicides mentioned above (Figure S8).

More importantly, the P450 inhibitor malathion could reverse the resistance to those three herbicides. Treatment with malathion (55 μM) had no response to the control line CK-GFP and three 77B34 lines. Synergism of herbicides and malathion could reduce the GR_50_ of three 77B34 lines, while it had no effect on CK-GFP (Table 1, Figure S6).

### 2.5. Absorption and Metabolism of Tribenuron-Methyl by Transgenic Arabidopsis Expressing CYP77B34

The Retention time of tribenuron-methyl was 1.366 min (Figure S9). The determination coefficients (R^2^) of two linear curves were 0.9951 (elution matrix) and 0.9914 (extract matrix) (Figure S10). Average recoveries were 90.93–105.19% (elution matrix) and 81.36–91.80% (extract matrix) at three different addition levels (Table S1). Relative standard deviation (RSD) of repeatability was below 5% (*n* = 4) (Table S1). The result showed that this method had high accuracy and good repeatability and was suitable for the absorption and metabolism analysis of tribenuron-methyl in *Arabidopsis*.

**Figure 3:**
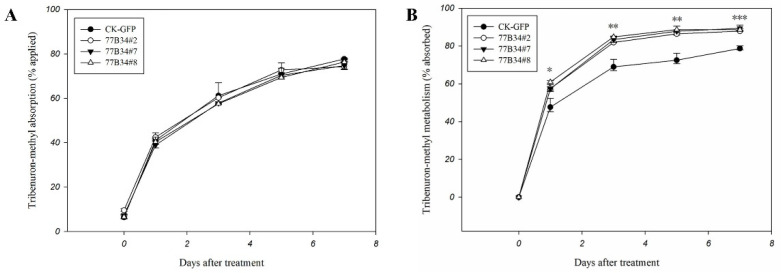
Tribenuron-methyl absorption (**A**) and metabolism (**B**) in control (CK-GFP) and transgenic *Arabidopsis* lines (77B34#2, 77B34#7 and 77B34#8). Each data point is the mean ± SE of four replicates. *, ** and *** indicate the mean of tribenuron-methyl metabolized in CK-GFP, and three 77B34 lines displayed significant differences at *p* < 0.05, 0.01 and 0.001, respectively.

The reduced dosage between what was applied to and eluted from leaf surface was considered the absorption dosage of tribenuron-methyl by transgenic *Arabidopsis* plants. The absorption of tribenuron-methyl by CK-GFP and 77B34 lines (77B34#2, 77B34#7 and 77B34#8) displayed no significant differences at 0, 1, 3, 5 and 7 DAT (Figure 3A).

The difference between the absorption and residue in transgenic *Arabidopsis* plants was counted as tribenuron-methyl metabolism. The metabolic rate of tribenuron-methyl in three transgenic lines (77B34#2, 77B34#7 and 77B34#8) was significantly higher than that in line CK-GFP at 1, 3, 5 and 7 DAT (Figure 3B). Three transgenic *Arabidopsis* lines exhibited no significant differences in tribenuron-methyl metabolism at 1, 3 and 5 DAT. However, the metabolic ability for tribenuron-methyl of 77B34#7 and 77B34#8 was significantly higher than that in 77B34#2 at 7 DAT (Table S2).

## 3. Discussion

The roles of P450s in herbicide metabolism and weed resistance are well-known and have been reviewed in detail elsewhere [7,26]. Nevertheless, most of the existing evidence on P450’s involvement in herbicide resistance is indirect according to the results of synergism of P450 inhibitors, RNA-seq, transcript level or contents of P450s [27,28,29]. The strongest evidence on P450 conferring herbicide resistance is the expressing P450 allele in a herbicide-sensitive individual or a substituted organism (such as *Arabidopsis*), and observing a decrease of susceptibility to herbicides [3]. However, identification of herbicide-metabolizing and resistance-endowing genes is slow, and only a few P450 genes have been identified in grass weeds of *Echinochloa phyllopogon*, *Echinochloa crus-galli* and *Lolium rigidum*. The metabolisms of these P450s to herbicides were confirmed by heterologous expression. For example, *Arabidopsis* or *Oryza sativa* L. expressing *E. phyllopogon* P450 genes (*CYP81A12, CYP81A14, CYP81A15, CYP81A18, CYP81A21, CYP81A24, CYP81A63*) display metabolic ability for herbicides of ALS-inhibiting herbicides (pyrazosulfuron-ethyl, bensulfuron-methyl, penoxsulam, pyriftalid, pyrimisulfan, propyrisulfuron, chlorsulfuron, azimsulfuron or propoxycarbazone-sodium), ACCase-inhibiting herbicides (diclofop-methyl, tralkoxydim, pinoxaden), Deoxy-D-xyulose phosphate synthase-inhibiting herbicide (clomazone), PSII-inhibiting herbicide (bentazone), HPPD-inhibiting herbicide (mesotrione), PPO-inhibiting herbicide (pyraclonil) or (and) PDS-inhibiting herbicide (norflurazon) [30,31,32,33]. *O. sativa* transferring the *E. crus-galli CYP81A68* gene has shown resistance to an ALS-inhibiting herbicide (penoxsulam) and ACCase-inhibiting herbicides (cyhalofop-butyl and metamifop) [34]. Another *O. sativa* transferring the *L. rigidum CYP81A10v7* gene became highly resistant to ACCase- and ALS-inhibiting herbicides (diclofop-methyl, tralkoxydim, chlorsulfuron) and moderately resistant to HPPD-inhibiting herbicide trifluralin [35].

We noticed that studies on metabolic herbicide resistance in broad-leaf weed species are limited, and genes related to herbicide metabolism almost remain unknown [24,36,37,38,39]. In recent years, our team has proved the important role of P450s in *D. sophia* resistance to tribenuron-methyl through a series of experiments, such as synergism of P450 inhibitors, absorption/metabolism, RNA- seq and qRT-PCR. A batch of metabolic enzyme genes including P450s (*CYP77B34, CYP709B8, CYP81F5, CYP96A146* and *CYP96A147*) were identified in TR *D. sophia* plants [24,25]. These P450s of *CYP77B34*, *CYP709B8, CYP81F5, CYP96A146* and *CYP96A147* may confer *D. sophia* resistance to tribenuron-methyl. Nevertheless, the function of a specific P450 gene in the metabolism and resistance to tribenuron-methyl is still ambiguous. In this work, the *CYP77B34* gene was transferred into *Arabidopsis*, and the sensitivities to tribenuron-methyl and other herbicides with different MoAs were observed. The results indicated that P450 of *CYP77B34* exhibited metabolic ability for herbicides with MoAs of an ALS-inhibiting herbicide (tribenuron-methyl), PPO-inhibiting herbicides (carfentrazone-ethyl and oxyfluorfen), a VLCFAs-inhibiting herbicide (pretilachlor) and a PSII-inhibiting herbicide (bromoxynil) (Figures S6 and S7). For three lines of *Arabidopsis* expressing *CYP77B34*, the RIs to tribenuron-methyl were 1.87, 3.08 and 2.17, respectively (Table 1). The P450 inhibitor malathion reduced the RI values of the three lines to 1.38, 1.62 and 1.67, which demonstrated that P450 (*CYP77B34*) mediated tribenuron-methyl metabolism (Table 1). In addition, tribenuron-methyl metabolism in transgenic *Arabidopsis* expressing *CYP77B34* was faster than that in CK-GFP (Figure 3). The present results indicated that *CYP77B34* mediated the metabolism and resistance to tribenuron-methyl in *D. sophia*. However, the proportion of resistant contribution caused by *CYP77B34* is unclear because of the enormous quantity of P450s and their unclear function in *D. sophia*. In the meantime, both the constitutive and induced expression levels of *CYP77B34* in TR *D. sophia* were significantly higher than that in TS *D. sophia* (Figure 1). This demonstrated that *CYP77B34* could be involved in resistance evolution to tribenuron-methyl in TR *D. sophia*. What we can not ignore is that the TR *D. sophia* had potential cross-resistance risk to carfentrazone-ethyl, oxyfluorfen, pretilachlor and bromoxynil due to its metabolism of these herbicides. Compared with those of TSR, the biggest threat is that NTSR causes weeds to evolve unpredictable cross-resistance to herbicides with different MoAs, even including herbicides that are not yet in the market. In addition, resistance management strategies of herbicide mixtures and rotations which are effective in managing TSR may have little or no effects on NTSR metabolic resistance [7,40]. This situation has been confirmed in many resistance cases mediated by P450. For example, *O. sativa* transferring the *L. rigidum CYP81A10v7* gene became highly resistant to ACCase- and ALS-inhibiting herbicides (diclofop-methyl, tralkoxydim, chlorsulfuron) and moderately resistant to HPPD-inhibiting herbicide trifluralin [35].

The evolution of new functions in plant metabolism usually experiences a very long process. However, the comparative analysis of plant P450s is helpful to understanding P450 function and evolution in plant metabolism [41]. Phylogenetic analysis of nucleotide sequences of *CYP77B34* was inferred using the neighbor-joining method employing MEGA7 software. The *CYP77B34* gene exhibited maximum similarity with members of theCYP77A and CYP77B subfamilies (Figure S3). The results of recombinant enzymes demonstrate that CYP77s can either in-chain hydroxylate or epoxidize fatty acids, in particular with 16- and 18-carbon chain lengths to form multiple precursors of cutin and suberin [42,43]. For example, *Arabidopsis CYP77A6* was confirmed to be involved in the synthesis of flower cutin monomers [44], and *Arabidopsis CYP77A4* is involved in signaling defense with epoxidation and in-chain hydroxylation [45,46]. *CYP77A1* and *CYP77B1* in petunia were related to the development of ornamental organs [42,47]. In P450-catalyzed herbicide metabolism, alkyl- or aryl-hydroxylation is one of the most common reactions [48]. Hydrophobic herbicide molecules can be oxidized to more hydrophilic metabolites by the hydroxylation reaction catalyzed by P450, which is the key step of herbicide degradation. *CYP77B34* may then play this pivotal role in the metabolism of herbicides. Meanwhile, the CYP77B34 protein is localized in the endoplasmic reticulum, which encompasses various kinds of molecular machines of proteins, including folding, quality control, signal transduction and degradation [49]. CYP77B34 may play one or more roles in that.

## 4. Materials and Methods

### 4.1. Plant Materials

The tribenuron-methyl-susceptible (TS) *D. sophia* population (SD8) was originally collected from remote areas at Linyi city of Shandong province in China (35°05′45.00″ N, 118°09′3.78″ E), and two tribenuron-methyl-resistant (TR) *D. sophia* populations (pHB23, N11) were harvested from winter wheat fields at Baoding city of Hebei province (38°48′55.4″ N, 115°23′23.0″ E for pHB23; 38°36′32.80″ N, 115°01′52.50″ E for N11) in 2013. All the seeds were purified by individual plant propagation to ensure the consistency of seeds’ genetic backgrounds. *D. sophia* of pHB23 and N11 evolved 258.3- and 116.3-fold resistance to tribenuron-methyl, respectively [25,50].

Seeds of *D. sophia* were sterilized with 20% H_2_O_2_ for 30 min, then soaked in a 0.3% gibberellin solution overnight after rinsing with distilled water. Next, these seeds were germinated on wet paper for 3–5 days in a climate chamber, then transplanted into plastic pots containing moist loam soil, then grown in a climate chamber under conditions of 25 °C/23 °C (day/night) temperature, 16 h photoperiod with light intensity of 20,000 lux.

*Arabidopsis thaliana* ecotype Columbia (Col-0) and *Nicotiana benthamiana* seeds were sterilized with 75% ethyl alcohol for 30 s and further sterilized in a 5% sodium hypochlorite solution for 8 min after rinsing with sterile water. Finally, the seeds were washed with sterile water and germinated on Murashige and Skoog solid medium (MS) for 2–3 weeks in a climate chamber with the same conditions as above after vernalization in 4 °C for 12–24 h [51]. These operations were performed under sterile conditions.

### 4.2. Expression Levels Determination of CYP77B34 in D. sophia

TS and TR *D. sophia* populations at the 6-leaf stage were treated with 15 μL tribenuron-methyl acetone solutions with concentration of 20 mg L^−1^ (0.3 μg per plant) by a micro applicator (Hamilton PB600 dispenser, Hamilton Co., Reno, NV, USA). The seedlings above the ground were collected for qRT-PCR at 0, 1, 3, 5 and 7 days after treatment (DAT). At each time point, four plants were harvested as one replicate, and three replicates were applied. Total RNA was extracted according to the instructions of an RNApre Pure Plant Kit (TIANGEN, Beijing, China). cDNA was synthesized using RNA as a template according to the instructions of a TIANScriptII RT Kit (TIANGEN, Beijing, China).

The expression levels were determined according to the instructions of a KAPA SYBR FAST qPCR Kit by an ABI Prism 7500 Real-Time PCR System (Applied Biosystems, Foster city, CA, USA). A total of 20 μL of reactions mixtures contained 10 μL 2 × KAPA SYBR FAST qPCR Master Mix Universal, 0.4 μL 50 × ROX Row, 0.6 μL primer pairs (10 μM), 1 μL cDNA template and 7.4 μL ddH_2_O. Primer information of *CYP77B34* and reference gene 18sRNA were listed in Table 2. The qPCR program was as follows: 95 °C for 3 min and 40 cycles of (95 °C for 3 s, 60 °C for 20 s and 72 °C for 32 s). Data was collected at the stage of 72 °C for 32 s, and melting analysis was carried out to verify the absence of nonspecific amplification at the end of the cycles. The data of gene expression was analyzed with 7500 Software v2.3 (Applied Biosystems, Foster City, CA, USA). Relative expression ratio (as 2^−ΔΔCt^) was calculated by the comparative C_T_ method, where ΔC_T_ = [C_T_ target gene-C_T_ internal control gene]. One-way analysis of variance (ANOVA) with Dunnett’s post-test (α = 5%) was conducted to assess significant differences by IBM SPSS Statistics 21 (International Business Machines Corporation, Armonk, NY, USA). Three biological replicates and four technical replicates were performed for each time point of TS and TR *D. sophia* populations [52].

### 4.3. Cloning and Phylogenetic Analysis

Five plants of TR *D. sophia* (N11) at the four-leaf stage were used for RNA extraction. Total RNA extraction and cDNAs synthesis were the same as in the above-mentioned methods. The amplification of the *CYP77B34* gene was performed using TIANSeq HiFi Amplification Mix (TIANGEN, Beijing, China). The PCR reaction mixtures contained 100 ng of cDNA, 0.25 μM of forward and reverse primers (Table 2) and 1 × HiFi amplification mix. The PCR conditions were as follows: 94 °C for 2 min, 35 cycles (98 °C for 10 s, 60 °C for 30 s and 68 °C for 45 s) and an extension step of 68 °C for 5 min. The PCR products were sequenced (Sangon Biotech, Shanghai, China) and aligned with DNAMAN 6.0 software (Lynnon Biosoft, San Ramon, CA, USA).

The phylogenetic analyses of *CYP77B34* were performed with the Mega 7 program using available nucleotide sequences of plant P450s in the CYP77 family by the neighbor-joining method [53]. Bootstrap analysis with 1000 replicates was conducted in order to obtain confidence levels for the branches. In order to facilitate and simplify the result, only major groups were shown.

### 4.4. pCAMBIA1302-CYP77B34-GFP Vector Construction and Agrobacterium Transformation

The cDNA of *CYP77B34* with a full-length sequence was inserted in the pCAMBIA1302-GFP vector with 35S promoters according to the instructions of a Clone ExpresII One-Step Cloning Kit (Vazyme Biotech, Nanjing, China). The pCAMBIA1302-CYP77B34-GFP vector was transformed into *Escherichia coli*, and the insert fragment sequence was confirmed by sequencing. The pCAMBIA1302-CYP77B34-GFP plasmid was purified and then transferred into the *Agrobacterium tumefaiciens* strain GV3101 (Biomed, Beijing, China).

### 4.5. Subcellular Localization

Tobacco leaves’ transient expression systems were used to determine the subcellular localization of the CYP77B34 protein. The subcellular localization was conducted according to the methods described by Pang et al. [54]. pCAMBIA1302-CYP77B34-GFP, the empty vector pCAMBIA1302-GFP and endoplasmic-reticulum (ER)-specific marker (mCherry-HDEL) were co-expressed in tobacco leaves. *A. tumefaciens* (GV3101 strain) transformed with pCAMBIA1302-CYP77B34-GFP, pCAMBIA1302-GFP or mCherry-HDEL was incubated at 28 °C with shaking (200 rpm) for 18 h. Bacteria were collected by centrifugation at 4000 rpm for 4 min and were resuspended to a final OD_600_ of 1.5 in an MES buffer (10 mM of MgCl_2_ and 10 mM of MES, pH 5.6) containing 150 nM of acetosyringone and were rested for 3 h. The bacteria suspensions mixed with a p19 silencing suppressor were infiltrated into leaves of *N. benthamiana*. Three days later, leaves of *N. benthamiana* were collected for microscopic observation using a Leica Stellaris5 confocal microscope (Leica, Heidelberg GmbH, Heidelberg, Germany). The green fluorescent protein (GFP) signal was excited at 488 nm and detected in the 500–550 nm range. The mCherry signal was excited at 561 nm and detected at 590–640 nm. The ImageJ plugin coloc 2 was used to measure Pearson’s correlation coefficient. More than 10 pictures were quantified for each co-localization.

### 4.6. Arabidopsis Transformation and Transcript Analysis of CYP77B34

*A. tumefaiciens*-transformed pCAMBIA1302-CYP77B34-GFP or pCAMBIA1302-GFP were transformed into *A. thaliana* by the standard floral dip method [55]. Transgenic *Arabidopsis* were selected on MS plates containing 25 μg mL^−1^ of hygromycin B, and homozygous plants were obtained by single plant reproduction for 3 consecutive generations. At least 4 independent transgenic lines, which was confirmed by PCR, were collected for each construct. The expression levels of *CYP77B34* in each line of transgenic *Arabidopsis* were determined by qRT-PCR.

Transgenic *Arabidopsis* at the 6-leaf stage was used for transcript analysis. The experiment was carried out on three biological and three technical replications. The GAPDH gene was selected as a reference gene, and the primer information is shown in Table 2. The qRT-PCR program was the same as that in expression levels’ determination of *CYP77B34* in *D. sophia*.

### 4.7. Herbicide Sensitivity of Transgenic Arabidopsis Expressing CYP77B34

The transgenic *Arabidopsis* was cultivated in MS solid medium containing herbicides (or/and 55 μM of malathion) with different concentrations and kept in a climate chamber under conditions of 25 °C/23 °C (day/night) temperature, 16 h photoperiod with light intensity of 20,000 lux. The plant growth or fresh weight of transgenic *Arabidopsis* were observed or weighed. These herbicides included inhibitors of ALS (tribenuron-methyl, imazamox and flumetsulam), VLCFAs (pretilachlor), EPSPS (glyphosate), GS (glufosinate ammonium), ACCase (diclofop-methyl), PSII (bromoxynil and atrazine), microtubule assembly (propyzamide and pendimethalin), auxin mimics (fluroxypyr), PPO (fomesafen, carfentrazone-ethyl and oxyfluorfen) and an unknown MoA (napropamide).

GR_50_, 50% inhibition of plant growth reduction, was calculated using the four-parameter log-logistic equation by SigmaPlot 12.0 (Systat Software Inc., San Jose, CA, USA).

$$y=C+\left(D-C\right)/\left[1+{\left(\frac{x}{GR_{50}}\right)}^{b}\right]$$

where *b* is the slope at *GR*_50_, *C* and *D* are the lower and upper limits, respectively. The resistance index (RI) was calculated by *GR*_50_ of the transgenic *Arabidopsis* expressing CYP77B34-GFP divided by that of the transgenic *Arabidopsis* expressing GFP to estimate the resistance levels.

### 4.8. Tribenuron-Methyl Metabolism Analysis in Transgenic Arabidopsis Using LC-MS

Transgenic *Arabidopsis* plants expressing GFP and *CYP77B34* were treated with tribenuron-methyl (0.16 μg/plant) at the 6-leaf stage. Above-ground parts of transgenic *Arabidopsis* plants were harvested at 0, 1, 3, 5 and 7 DAT. For each time point, five plants were harvested as one replicate per line, and four replicates were applied.

The residual tribenuron-methyl on the surfaces of transgenic *Arabidopsis* plants was washed with 8 mL of acetonitrile. The elution of acetonitrile was filtered with a 0.22 μm syringe filter, and tribenuron-methyl was quantified by LC-MS (Agilent 1290 InfinityII-6470, Palo Alto, CA, USA). Above-mentioned plant tissues were used for tribenuron-methyl metabolism measurement. The plants were ground into powder in liquid nitrogen and sonicated for 10 min with 5 mL of acetonitrile (with 1% acetic acid). After adding 0.5 g of NaCl, the homogenate was vibrated by a vortex for 2 min and centrifuged at 5000 rpm for 5 min. A 1 mL supernatant acetonitrile layer was added into a 2 mL centrifuge tube (containing 50 mg of anhydrous MgSO_4_, 25 mg of PSA and 7 mg of GCB) and vibrated by a vortex for 1 min. Then, the supernatant was filtered with a 0.22 μm syringe filter after centrifugation at 12,000 rpm for 3 min and analyzed by LC-MS.

The analysis of tribenuron-methyl was conducted by LC-MS with Agilent HPLC packed Shim-pack GIST C18 (50 × 2.1 mm i.d., 2 μm). The mobile phase was composed of 80% water and 20% acetonitrile, and the flow rate was 0.4 mL/min. Injection volume was 2 μL, and run time was 2.5 min. MS ran at conditions of DL temperature of 250 °C, heat block temperature of 300 °C, nebulizing gas flow of 5.0 L/min and drying gas flow of 11.0 L/min. The multiple reaction monitoring mode (MRM) for tribenuron-methyl was optimized at 396.2 > 155.1 under the above-mentioned conditions.

Linearity, recovery and precision were evaluated to ensure the quality of the analytical method. Linearity was determined by injecting tribenuron-methyl working standard solutions at concentrations of 0.001, 0.005, 0.01, 0.05, 0.1 and 0.2 mg/L followed by linear regression analysis. Precision was calculated as relative standard deviation (RSD) from recovery studies with standard spiked samples (*n* = 4) at levels of 0.01, 0.1 and 0.2 mg/L (for the elution matrix) and 0.016, 0.16 and 0.32 mg/L (for the extract matrix).

Data of tribenuron-methyl absorption and metabolism were subjected to ANOVA with Duncan’s test by IBM SPSS Statistics 21. Four replicates were conducted for each line.

## 5. Conclusions

In summary, CYP77B34 from *D. sophia* is a typical P450 protein localized in the ER. The constitutive and induced expression levels of the *CYP77B34* gene in TR *D. sophia* was significantly higher than that in TS plants. *Arabidopsis* expressing *CYP77B34* exhibited obvious resistance to tribenuron-methyl, carfentrazone-ethyl and oxyfluorfen. The P450 inhibitor malathion could reverse the transgenic *Arabidopsis* resistance to three herbicides. Compared to that of CK-GFP, three 77B34 lines possessed stronger metabolic ability for tribenuron-methyl. In addition, transgenic *Arabidopsis* expressing *CYP77B34* also displayed tolerance of bromoxynil and pretilachlor. The results provided direct evidence on *CYP77B34* endowing *D. sophia* resistance to tribenuron-methyl. At the same time, it also proved that cross-resistance patterns caused by P450 could be unpredictable.

## Figures and Tables

**Table 1:** Herbicide sensitivity of different transgenic *Arabidopsis* lines expressing *CYP77B34* to ALS-inhibiting herbicide (tribenuron-methyl) and PPO-inhibiting herbicides (carfentrazone-ethyl and oxyfluorfen) with or without malathion treatment.

Lines	Tribenuron-Methyl				Carfentrazone-Ethyl				Oxyfluorfen			
	−M ^a^		+M ^b^		−M		+M		−M		+M	
	GR_50_ (μM) ^c^	RI ^d^	GR_50_ (μM)	RI	GR_50_ (nM)	RI	GR_50_ (nM)	RI	GR_50_ (nM)	RI	GR_50_ (nM)	RI
CK-GFP	9.26 ± 2.08	1.00	9.00 ± 0.67	1.00	12.35 ± 1.00	1.00	11.39 ± 0.50	1.00	9.02 ± 1.36	1.00	10.02 ± 1.82	1.00
77B34#2	17.31 ± 1.31	1.87	12.45 ± 0.74	1.38	16.53 ± 0.74	1.34	13.10 ± 0.62	1.15	17.40 ± 0.77	1.93	13.36 ± 2.00	1.33
77B34#7	28.48 ± 1.96	3.08	14.61 ± 1.02	1.62	22.48 ± 1.01	1.82	14.07 ± 0.83	1.24	19.25 ± 0.52	2.13	12.60 ± 1.51	1.26
77B34#8	20.09 ± 1.87	2.17	15.27 ± 1.67	1.70	20.69 ± 0.90	1.68	12.91 ± 0.89	1.13	18.92 ± 1.34	2.10	11.55 ± 2.16	1.16

^a^ **−**M indicates treatment without malathion; ^b^ +M indicates treatment with malathion; ^c^ GR_50_, herbicide rate causing 50% growth reduction of plants; ^d^ RI, resistance index.

**Table 2:** Information of primers for cloning, vector construction and qRT-PCR of the *CYP77B34* gene.

Primer Pairs	Nucleotide Sequences [5′-3′]	Annealing (°C)	Products (bp)
Full length of *CYP77B34*			
CYP77B34-F	ATGGATCTTACCGACGTTATC	60	1530
CYP77B34-R	TCAAGTCCTTGACAGGATCTG		
pCAMBIA1302 construction for *Arabidopsis* transformation ^a^			
TY-77B34-F	AGAACACGGGGGACTCTTGACATGGATCTTACCGACGTTATCATATT	60	1572
TY-77B34-R	TTTACTAGTCAGATCTACCATAGTCCTTGACAGGATCTGGGC		
qRT-PCR			
PCR-77B34-F	TAAAGACGTTATGCTCATAAC	60	182
PCR-77B34-R	TCTCGTCGTTAGGACTCGCC		
18S-F	TAGTTGGTGGAGCGATTTGTCTG	60	114
18S-R	CTAAGCGGCATAGTCCCTCTAAG		
GAPDH-F	AGTCACTGTTTTCGGCATCA	60	139
GAPDH-R	AACTTTCTTGGCACCACCCT		

^a^ The underlined nucleotide sequence was the homologous sequence of the end of the linearized vector.

## Data Availability

Not applicable.

## Supplementary Materials

The following supporting information can be downloaded at: https://www.mdpi.com/article/10.3390/ijms23105812/s1.
