# Supplementary material for: The Metabolism of a Novel Cytochrome P450 (CYP77B34) in Tribenuron-Methyl-Resistant Descurainia sophia L. to Herbicides with Different Mode of Actions
Source: Int J Mol Sci. 2022 May 22;23(10):5812. doi: 10.3390/ijms23105812 (PMC9147942; doi:10.3390/ijms23105812)
Supplement: Supplementary file 1 [file ijms-23-05812-s001.zip › ijms-1741661-supplementary.pdf]

Article

The Metabolism of a Novel Cytochrome P450 (*CYP77B34*) in Tribenuron-Methyl-Resistant *Descurainia sophia* L. to Herbicides with Different Mode of Actions

Jing Shen, Qian Yang, Lubo Hao, Lingling Zhang, Xuefeng Li and Mingqi Zheng *

Department of Applied Chemistry, College of Science, China Agricultural University, Beijing 100193, China; shenjingtust@cau.edu.cn (J.S.); yangq1991@163.com (Q.Y.); 13156971873@163.com (L.H.); zhangll@cau.edu.cn (L.Z.); lixuefeng2007@163.com (X.L.)

* Correspondence: mqzheng@cau.edu.cn

Figure content:

Figure S1. Nucleotide and amino acid sequence of CYP77B34 from *Descurainia sophia* L.

Figure S2. Multiple sequence alignment of nucleotide sequences of *CYP77B34* with their respective homologs using DNAMAN software.

Figure S3. Phylogenetic analysis of nucleotide sequences of *CYP77B34* was inferred using the Neighbour-joining method employing MEGA7 software.

Figure S4. Pearson correlation coefficient between pCAMBIA1302-GFP and mCherry-HDEL or CYP77B34-GFP and mCherry-HDEL at *N. benthamiana* leaves.

Figure S5. Transcript levels of *CYP77B34* in transgenic *Arabidopsis*.

Figure S6. Dose-response curves of transgenic Arabidopsis expressed *CYP77B34* to tribenuron-methyl, carfentrazone-ethyl and oxyfluorfen, compared to the GFP control.

Figure S7. Susceptibility of *Arabidopsis* transformed with or without D. sophia CYP77B34 to tribenuron-methy, carfentrazone-ethyl, oxyfluorfen, bromoxynil and pretilachlor with different concentrations.

Figure S8. Sensitivities of transgenic *Arabidopsis* transformed with or without *D. sophia CYP77B34* to glyphosate, glufosinate ammonium, diclofop-methyl, imazamox, propyzamide, fluroxypyr, fomesafen, atrazine, flumetsulam, pendimethalin and napropamide with different concentrations.

Figure S9. Mass spectrogram of quantitative (A) and qualitative (B) ion in tribenuron-methyl.

Figure S10. Standard curve of tribenuron-methyl in elution matrix (A) and extract

matrix (B).

Table content:

Table S1. The recoveries of different addition levels of tribenuron-methyl in two matrixes.

Table S2. Tribenuron-methyl metabolized by different transgenic *Arabidopsis* plants at different time points.

```
1      ATGGATCTTACCGACGTTATCATATTCTTGTTCGCTCTCTACTTCATCAACCTATGGTGGCGCCGGTACTCCTCCGCCGGAAGCAGCCAGTGTCGCCTGAACATTCCACCGGGACCAAAA
1       M  D  L  T  D  V  I  I  F  L  F  A  L  Y  F  I  N  L  W  W  R  R  Y  S  S  A  G  S  S  Q  C  R  L  N  I  P  P  G  P  K

121    GGATGGCCACTCGTAGGTAACCTACTCCAAGTCATATTCCAACGTCGCCATTTTGTCTTCATAATGCGTGATCTACGTAAGAAGTTTGGTCCAATATTCACCATGCAAATGGGACAACGT
41      G  W  P  L  V  G  N  L  L  Q  V  I  F  Q  R  R  H  F  V  F  I  M  R  D  L  R  K  K  F  G  P  I  F  T  M  Q  M  G  Q  R

241    ACAATGATTATCGTCACGGACGAAAAACTCATCCACGAGGCGCTAGTTCAACGTGGCCCCACTTTCGCTTCCCGGCCCCCGGACTCCCCGATCCGACTCATGTTTAGCGTTGGAAAATGC
81      T  M  I  I  V  T  D  E  K  L  I  H  E  A  L  V  Q  R  G  P  T  F  A  S  R  P  P  D  S  P  I  R  L  M  F  S  V  G  K  C

361    GCTATAAACTCGGCGGAGTACGGATCTTTATGGCGAACTCTAAGGAGGAACTTCGTGACGGAGCTAGTGACCGCTACAAGAGTGAAGCAGTGCTCGTGGATACGGACTTGGGCTATGGAT
121     A  I  N  S  A  E  Y  G  S  L  W  R  T  L  R  R  N  F  V  T  E  L  V  T  A  T  R  V  K  Q  C  S  W  I  R  T  W  A  M  D
                                         Helix C
481    AATCATATGAAGAGGATTAAAACGGAGAATATTGAGAAAGGTTTCGTCGAGGTTATGAATCAATGCAGGTTAACGATCTGTAGCATCTTGATCTGTCTTTGTTTCGGCGCCAAGATCAGC
161     N  H  M  K  R  I  K  T  E  N  I  E  K  G  F  V  E  V  M  N  Q  C  R  L  T  I  C  S  I  L  I  C  L  C  F  G  A  K  I  S

601    GAGGGAAAGATTAAGAACATAGAGAATGTTCTTAAAGACGTTATGCTCATAACTTCCCCGACGTTACCGGACTTTTTGCCGGTATTCACTCCATTGTTTCGGCGTCAAGTACGAGAAGCG
201     E  G  K  I  K  N  I  E  N  V  L  K  D  V  M  L  I  T  S  P  T  L  P  D  F  L  P  V  F  T  P  L  F  R  R  Q  V  R  E  A

721    AGAGAGCTGAGGAAAACGCAACTCGAATGTCTTGTTCCGTTGATAAGAAACCGACGTAAGTTCGTTGATGCAAAGGCGAGTCCTAACGACGAGATGGTGAGTCCGATTGGTGCGGCTTAT
241     R  E  L  R  K  T  Q  L  E  C  L  V  P  L  I  R  N  R  R  K  F  V  D  A  K  A  S  P  N  D  E  M  V  S  P  I  G  A  A  Y

841    GTGGATTCTTTGTTTAATTTGAACCTGATCGAGAGAGGCGGTGAGCTTGGAGACGAAGAGATTGTGACTTTGTGTTCGGAAATTGTCTCGGCCGGTACGGACACGAGTGCAACGACGCTT
281     V  D  S  L  F  N  L  N  L  I  E  R  G  G  E  L  G  D  E  E  I  V  T  L  C  S  E  I  V  S  A  G  T  D  T  S  A  T  T  L
                                                                                                            Helix I
961    GAATGGGCGTTACTTCATCTAGTGACCGACCAAAACATTCAAGAGAAGCTATACGAGGAAGTCGTTGGAGTTGTTGGCAAAAACGGCATCGTTGAAGAGGATGACGTGGCAAAAATGCCT
321     E  W  A  L  L  H  L  V  T  D  Q  N  I  Q  E  K  L  Y  E  E  V  V  G  V  V  G  K  N  G  I  V  E  E  D  D  V  A  K  M  P

1081   TATTTGGAGGCTATTGTGAAAGAGACTCTCCGGCGACATCCTCCGGGACATTTCCTTCTCTCTCACGCTGCCGTGAAAGATACGGAGCTAGGAGGGTACGATATTCCGGCAGGGGCATAT
361     Y  L  E  A  I  V  K  E  T  L  R  R  H  P  P  G  H  F  L  L  S  H  A  A  V  K  D  T  E  L  G  G  Y  D  I  P  A  G  A  Y
                             Helix K
1201   GTGGAATTTTACACAGCTTGGGTTACTGAGAACCCGGAGATTTGGTCGGATCCGGGTAAGTTTCGACCCGAGAGGTTTTTAACCGGTGGGGACGGTGTTGATGCTGATTGGACCGGGACA
401     V  E  F  Y  T  A  W  V  T  E  N  P  E  I  W  S  D  P  G  K  F  R  P  E  R  F  L  T  G  G  D  G  V  D  A  D  W  T  G  T
                                                                   Meander domain
1321   CGTGGCGTTACAATGTTGCCTTTTGGTGCGGGTCGTAGGATCTGTCCGGCTTGGTCTTTGGGGATTCTACACATCAACTTGATGCTTGCTAGAATGATACATTCGTTTAAGTGGATCCCG
441     R  G  V  T  M  L  P  F  G  A  G  R  R  I  C  P  A  W  S  L  G  I  L  H  I  N  L  M  L  A  R  M  I  H  S  F  K  W  I  P
                               Heme binding domain
1441   GTTCCGGATTCTCCACCCGACCCGACTGAGACTTACGCATTCACGGTCGTGATGAAAAATTCTCTCAAAGCCCAGATCCTGTCAAGGACTTGA
481     V  P  D  S  P  P  D  P  T  E  T  Y  A  F  T  V  V  M  K  N  S  L  K  A  Q  I  L  S  R  T  *
```

Figure S1. Nucleotide and amino acid sequence of CYP77B34 from *D. sophia*. The *CYP77B34* gene contains a 1530-bp ORF encoding 510 amino acids. The underlined amino acid indicates the typical P450 conserved domains (Helix C, Helix I, Helix K, Meander domain and heme binding domain).

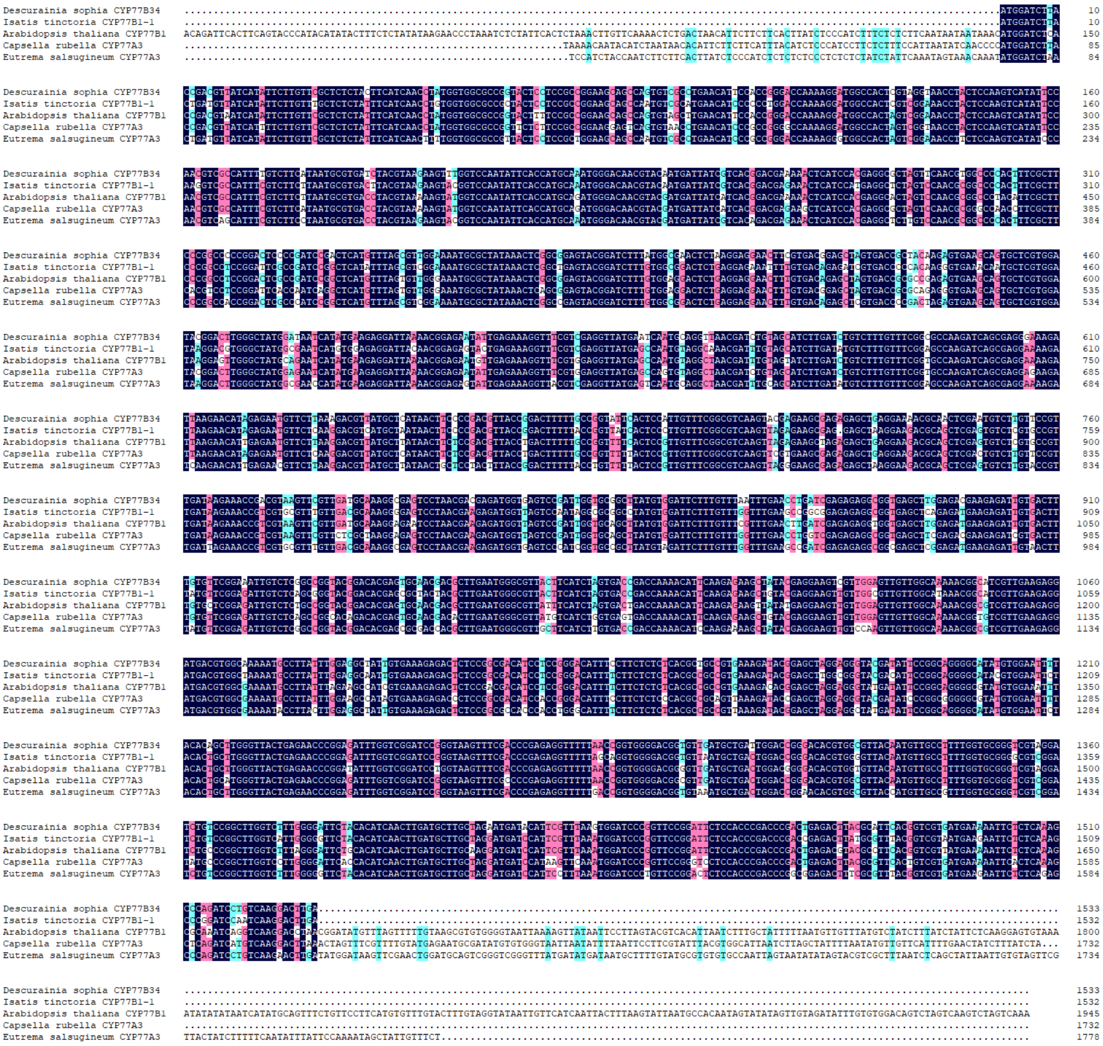

Figure S2. Multiple sequence alignment of nucleotide sequences of *CYP77B34* with their respective homologs using DNAMAN software. Sequences used for alignment were from *D. sophia* (*CYP77B34*: MF152623), *Isatis tinctoria* (*CYP77B1-1*: MT145859.1), *Arabidopsis thaliana* (*CYP77B1*: NM_101033.4), *Capsella rubella* (*CYP77A3*: XM_006307211.2) and *Eutrem sasugineum* (*CYP77A3*: XM_006417272.2).

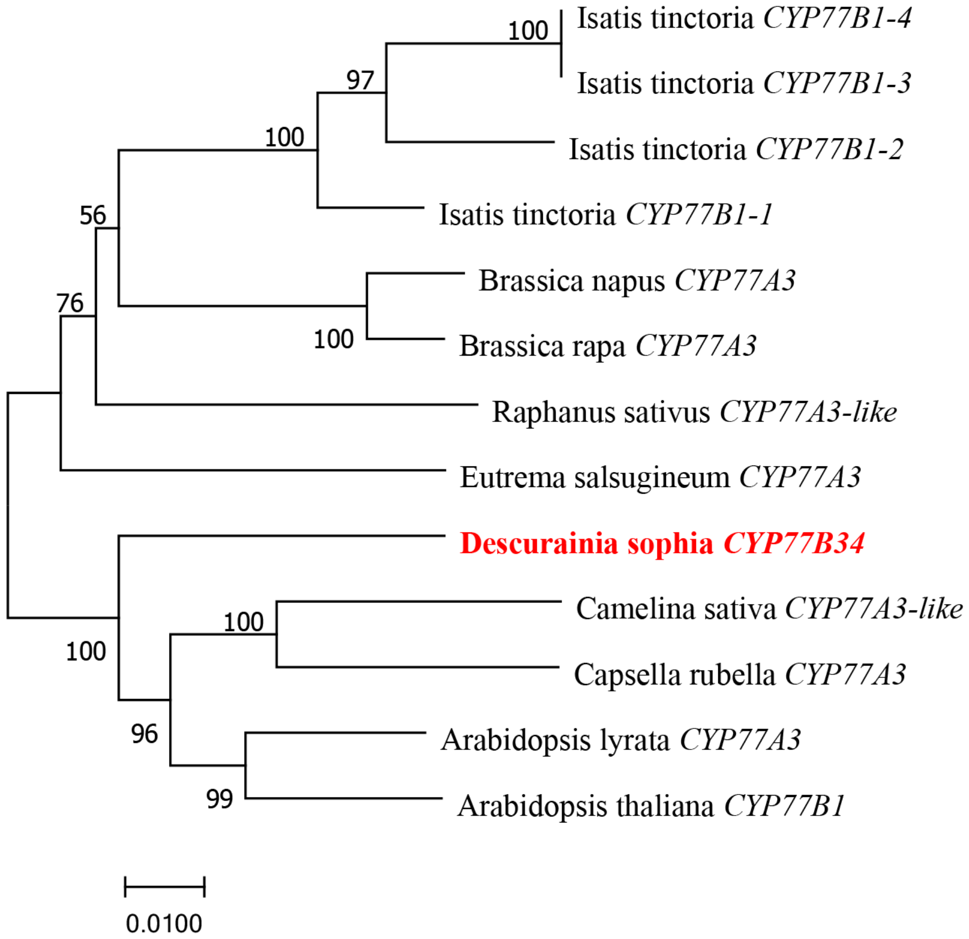

Figure S3. Phylogenetic analysis of nucleotide sequences of *CYP77B34* was inferred using the Neighbour-joining method employing MEGA7 software. Bootstrap analysis was performed with 1000 replicates. The number represents bootstrap values. Bar, 0.01 substitutions per site. Total 13 nucleotide sequences of *D. sophia* (MF152623), *I. tinctoria* (MT145859.1, MT145860.1, MT145881.1 and MT145882), *Brassia napus* (XM_013853378.2), *Brassica rapa* (XM_009119936.33), *Raphanus sativus* (XM_018594812.1), *E. salsugineum* (XM_006417272.2), *Cameline sativa* (XM_010494752.2), *C. rubella* (XM_006307211.2), *Arabidopsis lyrate* (XM_002889834.2) and *A. thaliana* (NM_101033.4) were used for analysis.

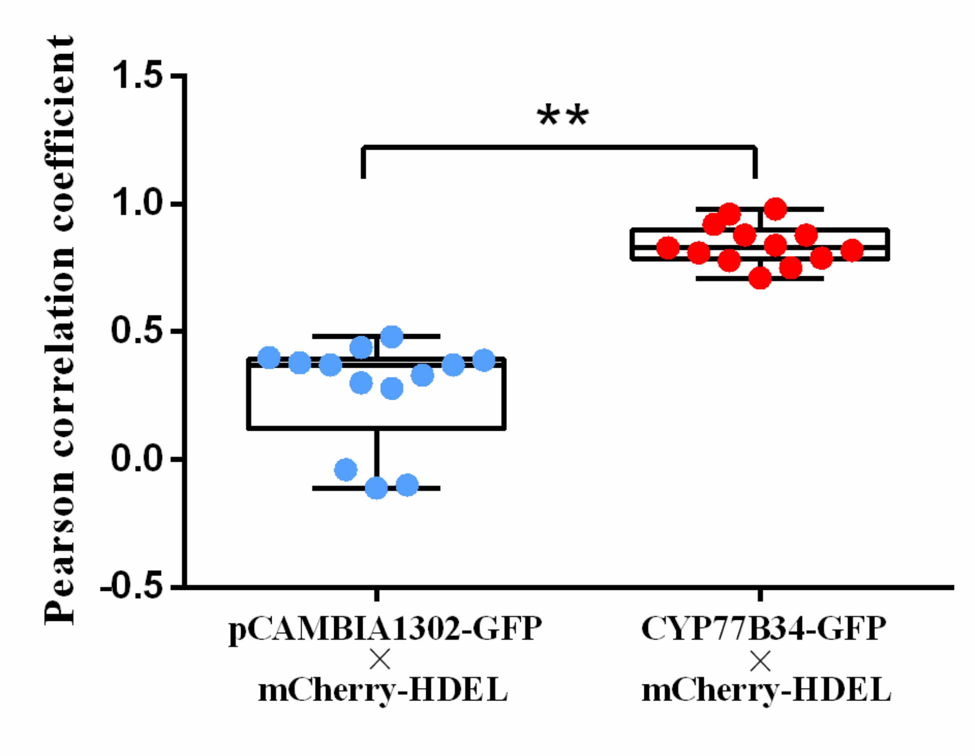

Figure S4. Pearson's correlation coefficient between pCAMBIA1302-GFP and mCherry-HDEL or CYP77B34-GFP and mCherry-HDEL at *N. benthamiana* leaves. n>10. Values shown are means ± SE. Significant differences were determined via one-way ANOVA with multiple comparisons. (**p⩽0.01)

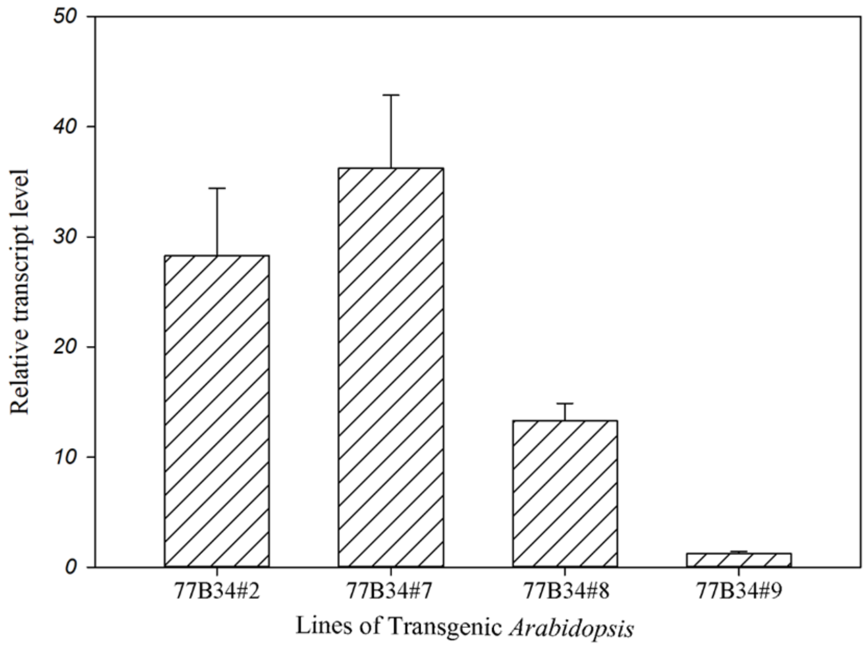

Figure S5. Transcript levels of *CYP77B34* in transgenic *Arabidopsis*. The expression levels of mRNA in different lines of transgenic *Arabidopsis* (77B34#2, 77B34#7, 77B34#8 and 77B34#9). GAPDH was the reference gene. Data was expressed as values relative to 77B34#9. The bars indicate the standard deviation.

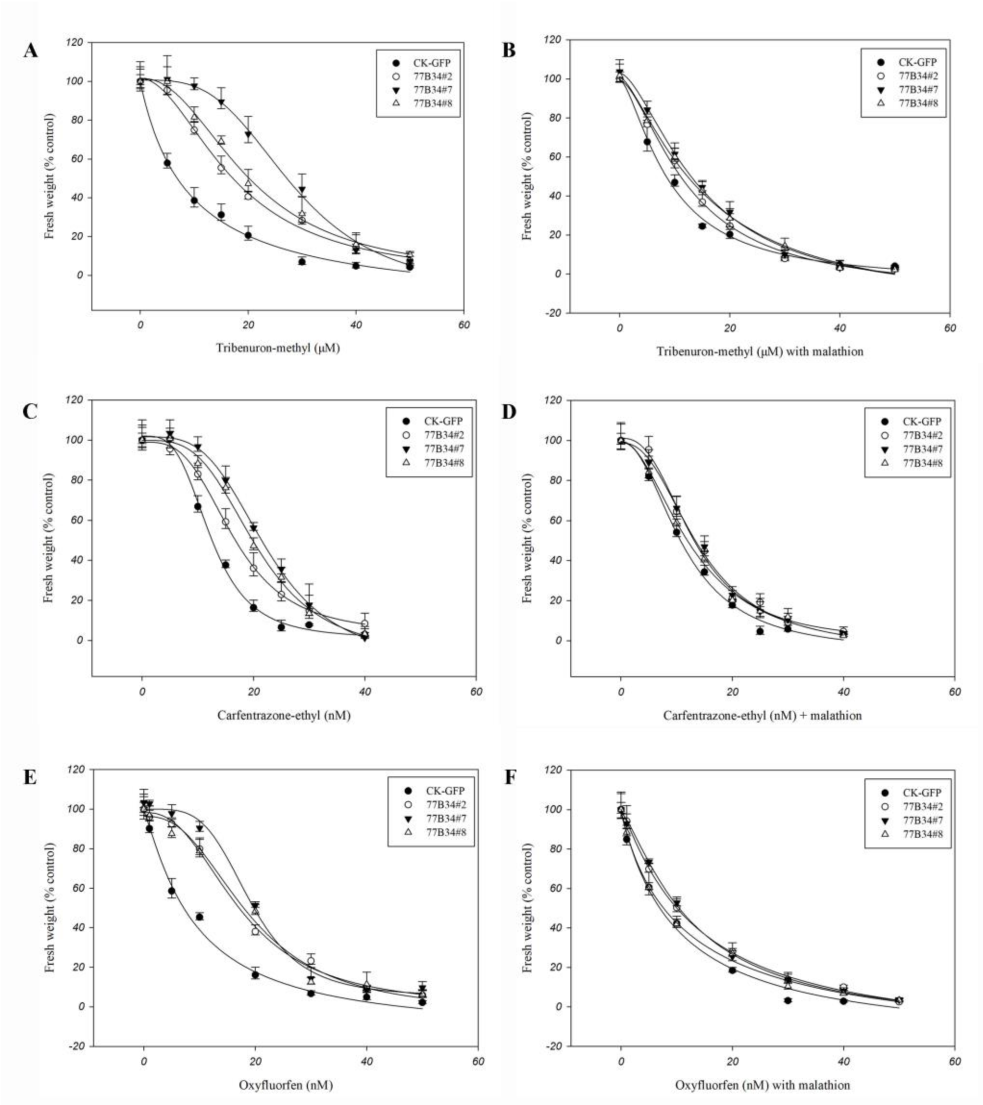

Figure S6. Dose-response curves of transgenic Arabidopsis expressed CYP77B34 (77B34#2, 77B34#7 and 77B34#8) to tribenuron-methyl (A and B), carfentrazone-ethyl (C and D) and oxyfluorfen (E and F), compared to the GFP control (CK-GFP). A, C and E were treated with herbicides; B, D and E were treated with herbicides and P450 inhibitor malathion (55 μM). Each data point is the mean ± SE of four replicates.

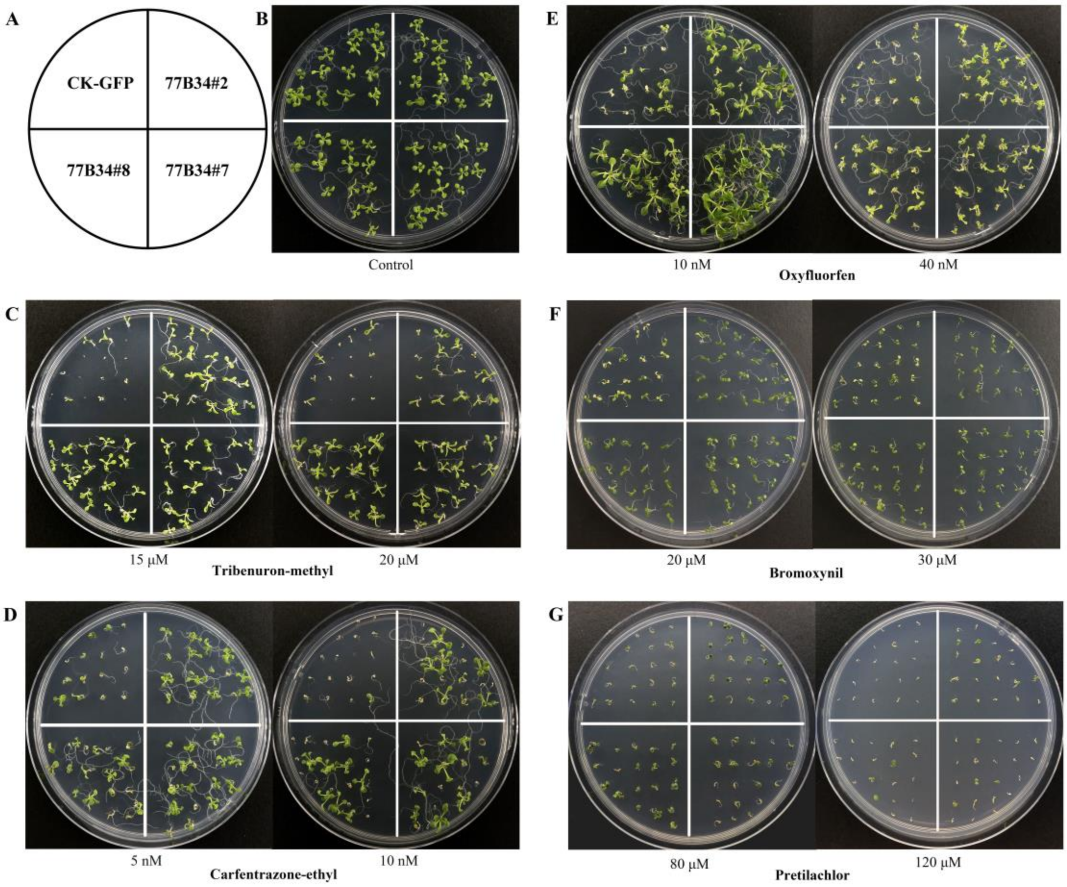

Figure S7. Susceptibility of *Arabidopsis* transformed with or without D. sophia CYP77B34 to tribenuron-methyl (C), carfentrazone-ethyl (D), oxyfluorfen (E), bromoxynil (F) and pretilachlor (G) with different concentrations. A: Distribution diagram of Arabidopsis carrying GFP or CYP77B34-GFP for all Petri dish. B: Transgenic Arabidopsis grows in medium without herbicide. Seedlings grown for 10 d on Murashige and Skoog (MS) medium. CK-GFP: transgenic Arabidopsis expressing empty plasmid; 77B34#2, 77B34#7 and 77B34#8: different lines of transgenic Arabidopsis expressing CYP77B34.

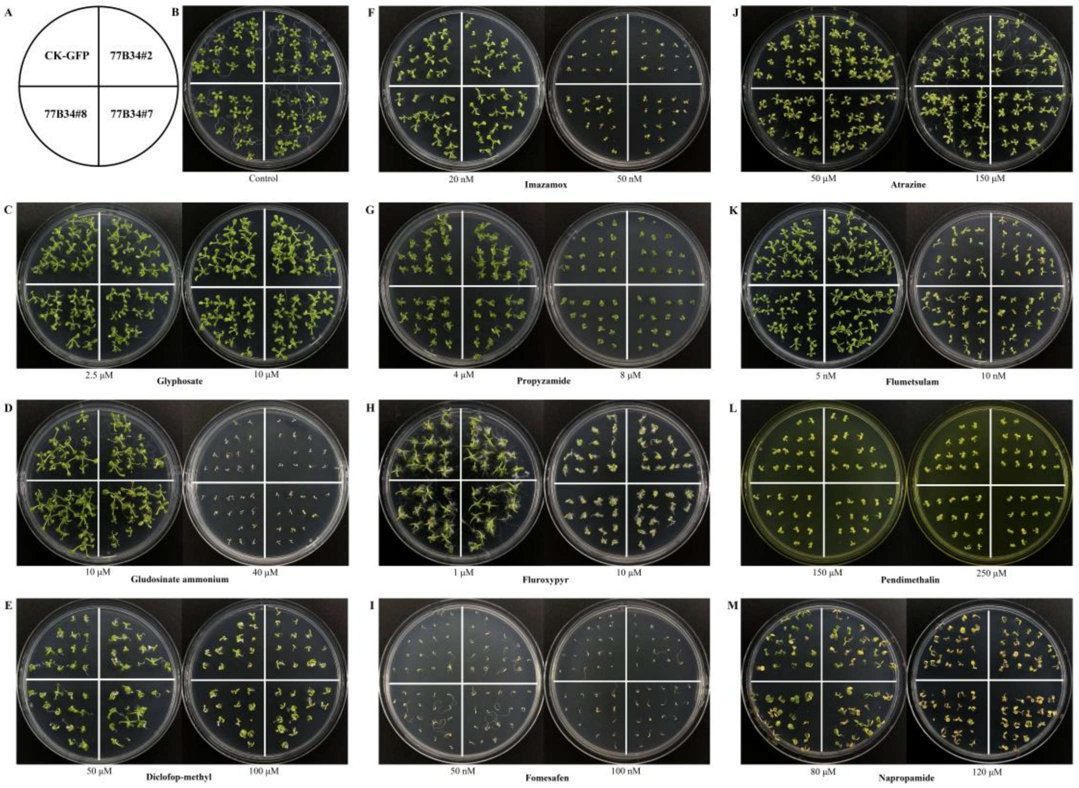

Figure S8. Sensitivities of transgenic *Arabidopsis* transformed with or without *D. sophia CYP77B34* to glyphosate (C), glufosinate ammonium (D), diclofop-methyl (E), imazamox (F), propyzamide (G), fluroxypyr (H), fomesafen (I), atrazine (J), flumetsulam (K), pendimethalin (L) and napropamide (M) with different concentrations. A: Distribution diagram of *Arabidopsis* carrying GFP or *CYP77B34*-GFP for all Petri dish. B: Transgenic *Arabidopsis* grows in medium without herbicide. Seedlings grown for 10 d on MS medium. CK-GFP: transgenic *Arabidopsis* expressing empty plasmid; 77B34#2, 77B34#7 and 77B34#8: different lines of transgenic *Arabidopsis* expressing *CYP77B34*.

A

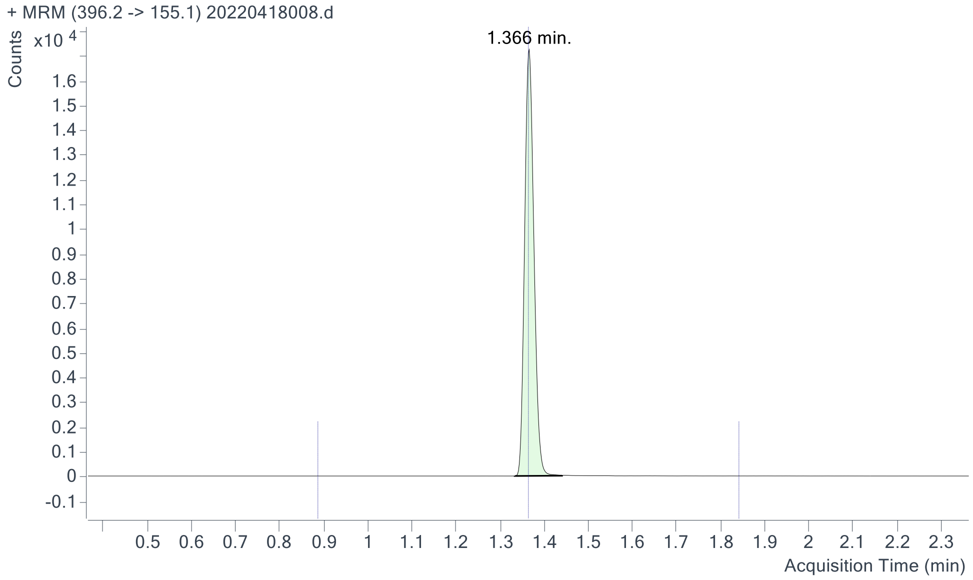

B

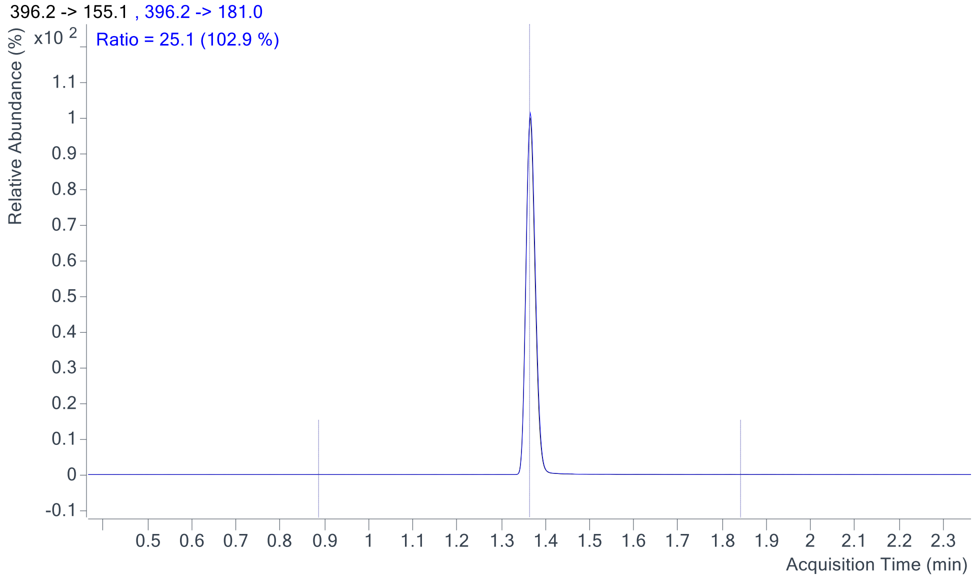

Figure S9. Mass spectrogram of quantitative (A) and qualitative (B) ion in tribenuron-methyl.

A

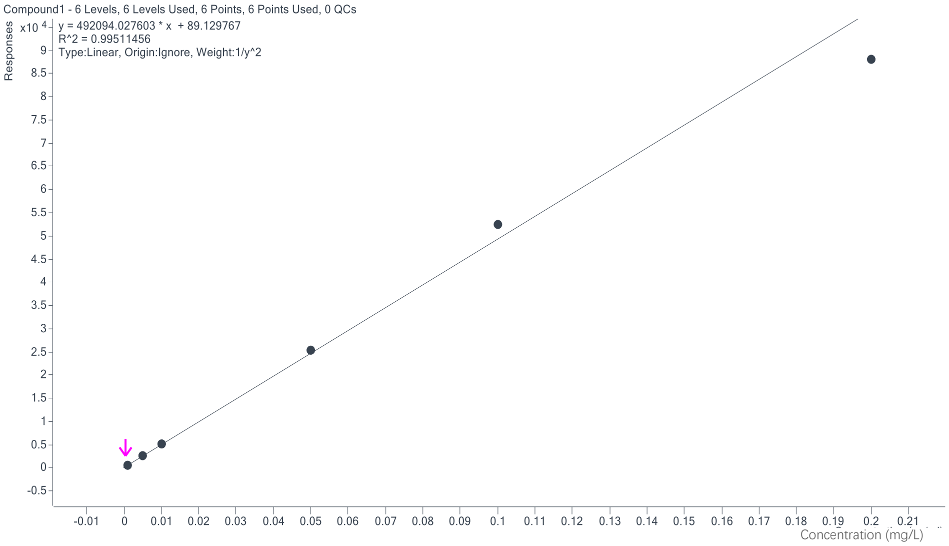

B

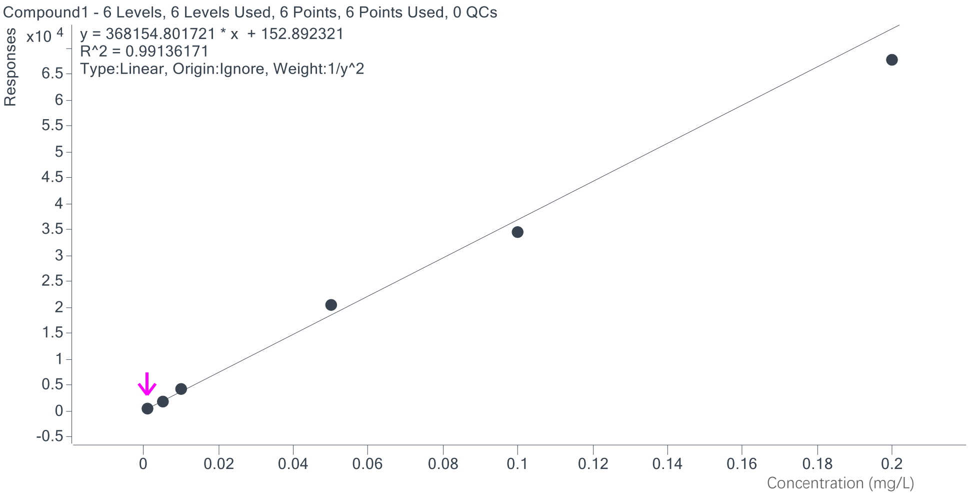

Figure S10. Standard curve of tribenuron-methyl in elution matrix (A) and extract matrix (B).

Table S1. The recoveries of different addition levels of tribenuron-methyl in two matrixes

Elution matrix			Extract matrix		
Addition levels (mg/L)	Recovery (%)	RSD (%)	Addition levels (mg/L)	Recovery (%)	RSD (%)
0.01	102.27	1.15	0.016	90.38	3.14
0.10	105.19	1.16	0.16	91.80	0.28
0.20	90.93	0.70	0.32	82.36	2.93

Table S2. Tribenuron-methyl metabolized by different transgenic *Arabidopsis* plants at different time points

Days after treatment	Tribenuron-methyl metabolism (% of absorption) [a]			
	CK-GFP	77B34#2	77B34#7	77B34#8
1	47.64±4.70a	56.38±3.25b	59.58±2.43b	57.16±3.01b
3	68.96±4.00a	82.13±0.43b	85.05±0.49b	83.33±1.07b
5	72.51±3.69a	86.37±1.10b	88.77±0.84b	87.06±0.28b
7	78.71±1.38a	87.57±0.88b	89.88±1.09c	89.05±0.15bc

[a] Rows with different letters indicate tribenuron-methyl metabolism are significant different (from SPSS analysis). Each data is mean ± SE of four replicates.
